# Quantitative assay of targeted proteome in tomato trichome glandular cells using a large-scale selected reaction monitoring strategy

**DOI:** 10.1186/s13007-019-0427-7

**Published:** 2019-04-24

**Authors:** Ayako Takemori, Taiken Nakashima, Hisashi Ômura, Yuki Tanaka, Keisuke Nakata, Hiroshi Nonami, Nobuaki Takemori

**Affiliations:** 1 Department of Bioresource Production Science, The United Graduate School of Agricultural Sciences, Ehime University, Matsuyama, 790-8566 Japan; 2 Research Faculty of Agriculture, Hokkaido University, Sapporo, 060-8589 Japan; 3 Graduate School of Biosphere Science, Hiroshima University, Higashi-Hiroshima, 739-8528 Japan; 4 Advanced Research Support Center, Ehime University, Toon, 791-0295 Japan; 5 Plant Biophysics/Biochemistry Research Laboratory, Faculty of Agriculture, Ehime University, Matsuyama, 790-8566 Japan; 6 Division of Proteomics Research, Proteo-Science Center, Ehime University, Toon, 791-0295 Japan

**Keywords:** Tomato type VI glandular trichome, Secondary metabolism, Plant defense mechanism, Targeted proteomics, Mass spectrometry, Selected reaction monitoring, Proteotypic peptide, Protein quantification, AQUA peptide, QconCAT

## Abstract

**Background:**

Glandular trichomes found in vascular plants are called natural cell factories because they synthesize and store secondary metabolites in glandular cells. To systematically understand the metabolic processes in glandular cells, it is indispensable to analyze cellular proteome dynamics. The conventional proteomics methods based on mass spectrometry have enabled large-scale protein analysis, but require a large number of trichome samples for in-depth analysis and are not suitable for rapid and sensitive quantification of targeted proteins.

**Results:**

Here, we present a high-throughput strategy for quantifying targeted proteins in specific trichome glandular cells, using selected reaction monitoring (SRM) assays. The SRM assay platform, targeting proteins in type VI trichome gland cells of tomato as a model system, demonstrated its effectiveness in quantifying multiple proteins from a limited amount of sample. The large-scale SRM assay uses a triple quadrupole mass spectrometer connected online to a nanoflow liquid chromatograph, which accurately measured the expression levels of 221 targeted proteins contained in the glandular cell sample recovered from 100 glandular trichomes within 120 min. Comparative quantitative proteomics using SRM assays of type VI trichome gland cells between different organs (leaves, green fruits, and calyx) revealed specific organ-enriched proteins.

**Conclusions:**

We present a targeted proteomics approach using the established SRM assays which enables quantification of proteins of interest with minimum sampling effort. The remarkable success of the SRM assay and its simple experimental workflow will increase proteomics research in glandular trichomes.

**Electronic supplementary material:**

The online version of this article (10.1186/s13007-019-0427-7) contains supplementary material, which is available to authorized users.

## Background

Approximately 30% of vascular plants have epidermal protrusions called trichomes on their aerial parts [1]. Multicellular trichomes that produce and store various secondary metabolites are classified as glandular-type trichomes [2, 3]. Glandular trichomes are commonly composed of the base, stalk, and head units, and are further classified into several subtypes based on their morphology. The glandular trichomes have one or more secretory cells, called trichome glandular cells (TGCs), on the head, and various types of secondary metabolites including terpenoids, flavonoids, and alkaloids are synthesized in TGCs [4–9]. TGCs rapidly secrete intracellular components in response to external physical stimulation, and the secreted components play important roles, such as plant defense against insects and pathogens [10], protection against desiccation [11], and as a barrier against atmospheric oxidative stress [12]. Glandular trichomes are called natural cell factories because of their production of special metabolites, and they have high commercial value as food and pharmaceutical products [13–15].

Several molecular omics studies have been conducted to better understand metabolite production processes in TGCs [16–20]. In particular, mass spectrometry (MS)-based analytical techniques have been used for highly sensitive and high-throughput detection of cellular molecules in TGCs. MS systems combined with separation techniques, e.g., gas chromatography [20, 21] or liquid chromatography (LC) [22], are used extensively to gain insights into the metabolomic profiles of various TGCs. Besides the analysis of cellular metabolomes, the effectiveness of MS has also been demonstrated in large-scale analyses of proteins related to metabolite production in TGCs [23–28]. Current proteomics research is mostly based on an experimental approach using an LC-tandem MS (MS/MS), called shotgun proteomics [29]. Shotgun proteomics is performed by sequencing the peptides derived from the protease digestion of proteins in biological samples, enabling large-scale identification of protein components. However, the complexity of the cellular proteome is high, and it is generally difficult to detect trace components reproducibly using shotgun proteomics. The amount of sample used for single shotgun analysis depends on the capacity of the LC–MS system, but a minimum of 10 μg of total protein as starting material is generally required to identify more than 100 proteins. Therefore, for in-depth proteome analysis, it is necessary to recover a large amount of TGCs, which makes the analysis difficult when the available sample is limited.

In addition to the shotgun approach, which aims to analyze as many proteins as possible, an alternative approach to quantify only the targeted proteins with minimum steps in a sensitive manner is required in TGC proteomics research. In this study, we focused on a novel MS-based proteomics strategy using selected reaction monitoring (SRM), also known as multiple reaction monitoring (MRM), to obtain accurate quantitative information of target proteins from a small amount of TGC sample. SRM has high sensitivity in terms of selectively quantifying target molecules in complex analytical samples [30]. Currently, for protein quantification, the LC-SRM assay using triple quadrupole MS combined with LC is used; with this approach multiple target proteins can be simultaneously detected with a single LC run [31–34]. Quantitative analysis using a large-scale SRM assay covering a group of cellular proteins involved in a specific biological process is now called “targeted” proteomics [35, 36], and its effectiveness has been demonstrated in various model organisms [37–40]. Here, we established an experimental workflow to develop a large-scale SRM assay for monitoring the TGC proteome and implemented targeted proteomics studies. We selected cultivated tomato *Solanum lycopersicum*, for which high-quality genome sequence information is available [41], and analyzed TGCs for type VI trichomes, which is the most abundant type in tomato [3]. By using an established workflow, comprehensive quantitative information on the targeted proteome can be easily acquired using a small amount of TGC sample. In the present study, we assessed whether established SRM assays could be used for comparative proteomic analysis of TGCs in three different tomato organs. Further, we evaluated whether a combination of established assays and internal standards could be used to quantify the absolute amounts of multiple target proteins in TGCs.

## Results

### Experimental strategies for SRM assay development

**Fig. 1:**
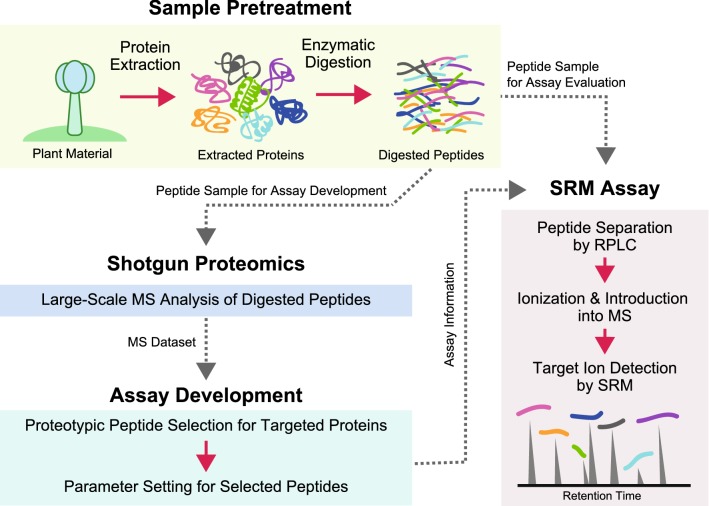
Schematic overview of the development of selected reaction monitoring (SRM) assays. All mass spectrometry (MS) analyses were conducted using a triple quadrupole/linear ion trap hybrid MS instrument connected with nanoflow reversed-phase liquid chromatography (RPLC). First, the glandular cell samples collected from the target trichome were subjected to a shotgun proteomics analysis. Based on proteomics information, suitable peptides for an SRM experiment were selected and SRM assays targeting selected peptides were designed. The established assay performance was assessed using the rest of the peptide samples used in the shotgun analysis

Figure 1 shows the workflow of the SRM assay developed to target TGC proteins. In the SRM assay, the biological samples are fragmented by digestion with a site-specific protease, trypsin or Lys-C, and the derived peptides are used in the SRM assay. The mixture of digested peptides is primarily separated by LC, followed by high selective detection of the peptides derived from the target proteins by quadrupole MS filters based on their mass to charge ratio. Therefore, the selection of target peptides with high ionization efficiency and reproducible detection, called “proteotypic peptides” [42], is indispensable for developing reliable assays. Due to the difficulty of theoretical prediction, it is desirable to select proteotypic peptides based on the datasets from shotgun proteomics analysis. In this study, we first conducted a shotgun proteomics analysis of TGCs by a gel electrophoresis-assisted LC–MS/MS (GeLC–MS/MS) approach [43] and surveyed the peptide candidates suitable for high-throughput SRM. Based on the obtained peptide information, we next established a large-scale SRM assay targeting TGC proteins with proteotypic peptides.

### GeLC–MS/MS analysis of TGC proteomes

**Fig. 2:**
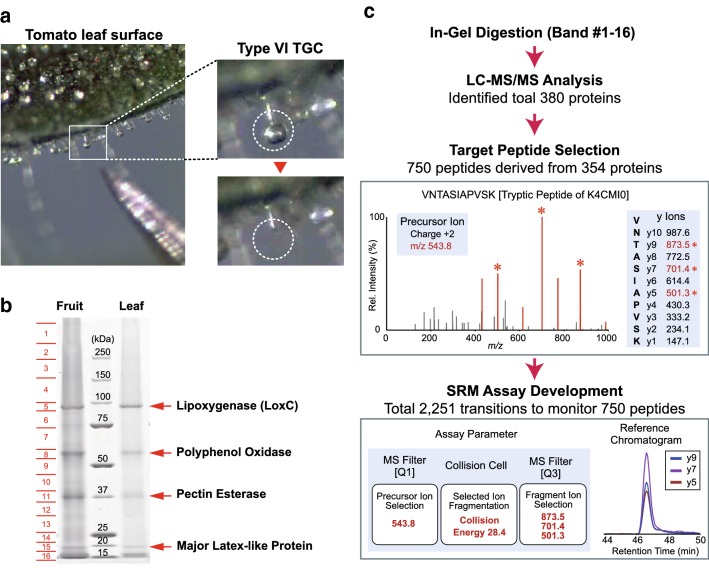
Development of a large-scale selected reaction monitoring (SRM) system targeting the tomato trichome gland cell (TGC) proteome. **a** Images of the TGC sampling procedure. The target TGCs from the head of type VI trichomes of tomato were manually recovered with a pair of fine forceps under a stereoscopic microscope. **b** Polyacrylamide gel electrophoresis (PAGE) of the TGC proteins. After sampling TGCs from 800 trichomes, cellular protein components were extracted. Protein extracts were separated using a 4–12% NuPAGE gel and protein bands were visualized with Bio-safe CBB. **c** Experimental workflow of the SRM assay development. Proteins separated by PAGE were digested in the gel; the resulting peptide digests were analyzed by liquid chromatography–mass spectrometry (LC–MS) and their sequences were identified by a database search. Based on the obtained MS information, the combination of a precursor ion and the fragment ions (SRM transition) was selected. Finally, for monitoring 750 peptides, 2 251 transitions were chosen in this study

Shotgun proteomics analysis was performed using glandular cells from type VI trichomes of tomato collected from different organs (the fruits and leaves). TGCs were collected manually under a stereo microscope using a pair of forceps (Fig. 2a). TGCs were stably placed at the tip of the forceps, enabling easy recovery of cellular components by immersing the tip rapidly in sodium dodecyl sulfate (SDS) lysis buffer. The amount of protein contained in glandular cells/trichomes was different among the organs; for example, 2.3 ng in the leaf and 6.1 ng in the green fruit (Additional file 1: Figure S1). In the GeLC–MS/MS analysis, the sample amount required for fractionation by SDS polyacrylamide gel electrophoresis (SDS-PAGE) was approximately 5 μg, which was obtained by TGC sampling from 800 glandular trichomes. The extracted TGC proteins were separated by SDS-PAGE and visualized by Coomassie blue staining (Fig. 2b). The SDS-PAGE gel image showed four major protein bands in the sample lane; the band at ~ 90 kDa was lipoxygenase (LoxC), known as a type VI TGC-specific protein [23]. The sample lane was divided into 16 pieces and in-gel protein digestion using trypsin was carried out with each gel piece. Half of the digested peptides were subjected to LC–MS/MS with data-dependent acquisition. As a result of a database search with the MS/MS data obtained, 380 proteins were identified from fruit TGCs (Additional file 2: Table S1). A total of 168 proteins were identified to be involved in cellular metabolic processes (GO: 0008152 and GO: 0044237) (Additional file 3: Figure S2). Many proteins (34) involved in response to stimulation (GO: 0050896) were also observed, as expected from the physiological function of the glandular trichomes.

### Assay development for the targeted proteins

Among the identified peptides from shotgun proteomics, we selected 750 peptides derived from 354 proteins as the target candidates for the SRM assays (Fig. 2c). In the MS/MS analysis, many digested peptides were generally observed from high-abundance proteins. On the contrary, only 1–3 peptides were observed from low-abundance proteins. Therefore, the number of peptides that can be monitored by SRM varies depending on the protein. In this study, up to five peptides per protein were selected as candidates. Peptides containing methionine residues are known to be unsuitable for quantitative analysis due to their oxidative modification. Therefore, peptide sequences containing methionine were excluded as much as possible. We then developed the SRM assays targeting peptide candidates. In the SRM assay, the combination of a precursor ion and the fragment ions, called SRM transition, enables the detection of targets with high specificity (Fig. 2c). In this study, we used Skyline software [44] to design 2251 transitions to monitor 750 peptides based on the MS/MS spectral information, and a large-scale SRM assay was developed using a triple quadrupole MS system connected online to a nanoflow LC. At least three transitions were set for monitoring the targeted peptide ion in the SRM assay. The workability of the established assays was verified using the remaining half of the in-gel digested peptide sample. Among the transitions designed in this study, 1587 transitions targeting 298 proteins (Additional file 4: Table S2) had peptide ion peaks with sufficient strength to be successfully detected.

### Targeted TGC proteomics using SRM

**Fig. 3:**
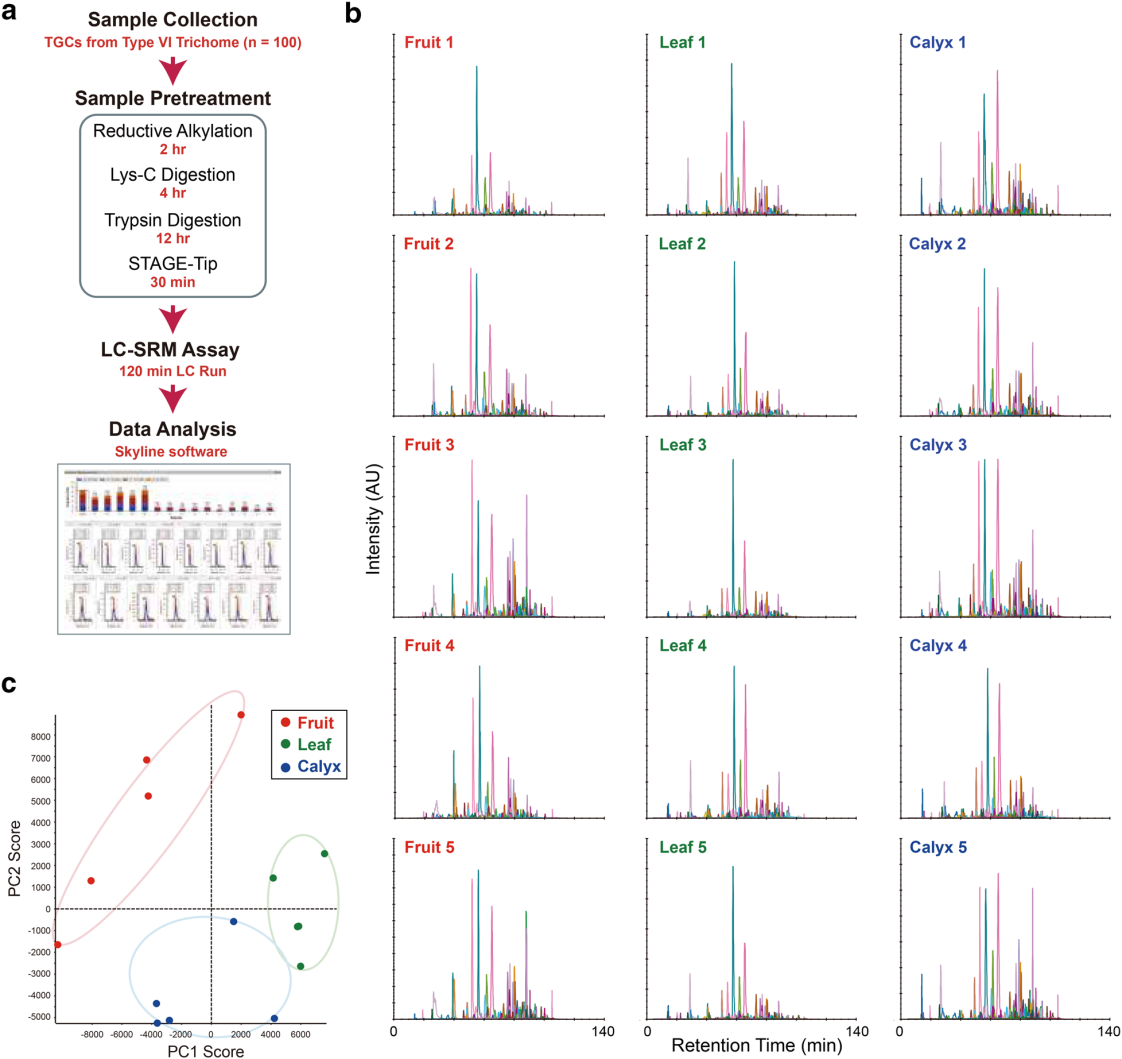
Targeted tomato trichome gland cell (TGC) proteomics by large-scale selected reaction monitoring (SRM) quantification. **a** Experimental workflow of tomato TGC proteomics using SRM. TGCs of type VI trichomes from tomato were recovered from different organs (the green fruits, leaves, and calyx). TGCs were collected directly into urea lysis buffer, and then subjected to an in-solution digestion treatment with trypsin/Lys-C. The peptide ions of interest were selectively monitored by the liquid chromatography (LC)-SRM assay and the peak intensities of the detected ions of the different organs were compared using Skyline software. **b** SRM profiles of the targeted TGC proteome in the different organs. Five biological replicates were used for each organ type. In our SRM system, 221 proteins were quantified. **c** Principal component analysis was performed based on the peak intensity of the peptides identified in the five replicates

**Fig. 4:**
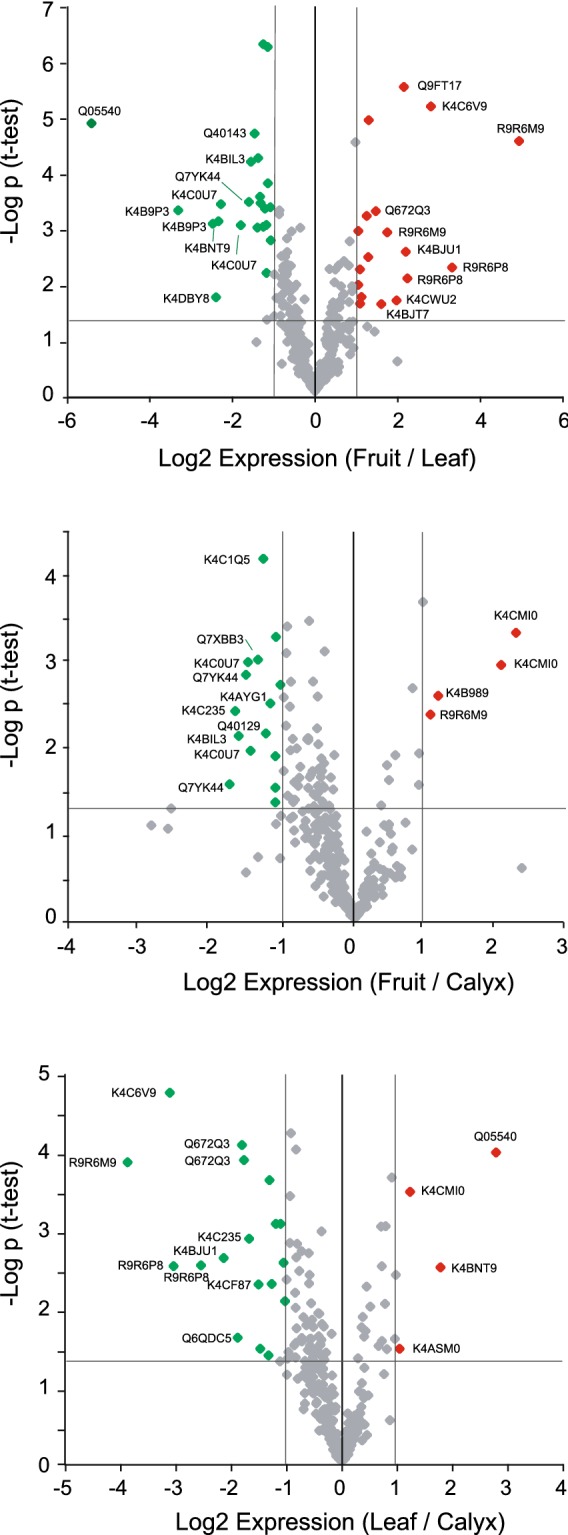
Volcano plots representing tomato trichome gland cell (TGC) protein expression profiles in three different organs of tomato. In the relative quantification with label-free selected reaction monitoring (SRM) assays, 331 peptides representing 221 proteins were quantified and used for *t*-tests to generate the volcano plot indicating the log2 expression ratio on the X-axis and − log *p* value on the Y-axis for each peptide. Detailed information of organ-enriched proteins is shown in Additional file 6: Table S4

In general, nanoflow LC-SRM can detect targeted proteins with high sensitivity from a smaller amount of sample than that used for LC–MS/MS analysis, which reduces the sample amount used for a single experiment. However, conventional sample preparation for shotgun proteomics using LC–MS/MS (assuming hundreds of micrograms) has a risk of sample loss and is not suitable for small sample volumes. Therefore, in this study we developed a sample pretreatment method suitable for trace amounts of TGCs. The experimental workflow for our sample pretreatment is shown in Fig. 3a. TGCs recovered using a pair of forceps were directly collected in 8 M urea solution and the protein sample extracted by sonication was subjected to protease digestion with trypsin and Lys-C. To reduce sample loss, all processes were completed in a single low-adsorption tube. To evaluate the established method, sample pretreatment for SRM was performed using TGCs that were recovered from green tomato fruits. We were able to recover ~ 400 TGCs from 100 trichomes in 5–10 min, and the recovery of TGC samples necessary for all experiments with five replicates was completed within 60 min. We performed the SRM assay using half of the obtained peptide samples and succeeded in detecting 221 proteins within a 120-min LC run (Fig. 3b). Using the established system, we further performed a comparative quantitative analysis of targeted proteome levels in TGCs derived from different organs (the leaves, green fruits, and calyx) of tomato (Fig. 3b and Additional file 5: Table S3). Each biological replicate contained TGCs collected from 100 trichomes. Principal component analysis (PCA) using the quantitative data obtained revealed organ-specific differences in TGC proteomes (Fig. 3c). Statistical analysis showed that proteins related to plant defense mechanisms, secondary metabolic processes, and anti-oxidative stress response are differentially expressed (Fig. 4 and Additional file 6: Table S4).

### Quantitative strategies using stable-isotope dilution MS

**Fig. 5:**
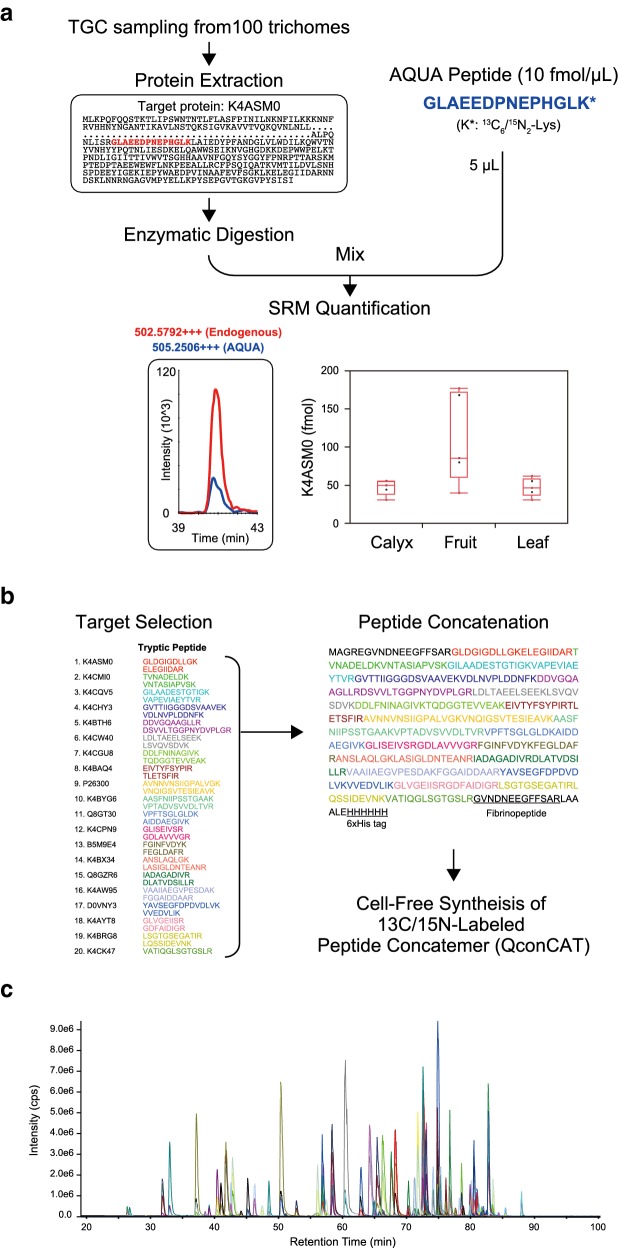
Selected reaction monitoring (SRM)-based quantitative strategies of targeted protein amount in tomato trichome gland cells (TGCs). **a** SRM determination of target protein abundance in the TGC sample using an AQUA peptide standard. A stable isotope-labeled AQUA peptide (GLAEEDPNEPHGLK) was used as an internal standard for the absolute quantification of the targeted K4ASM0 protein by SRM. **b** Biosynthesis workflow for the QconCAT peptide standards. Two QconCAT sequences covering 50 proteins involved in the synthesis of secondary metabolites (detailed information can be found in Additional file 8: Figure S4 and Additional file 9: Table S5) were designed in this study. Stable isotope-labeled QconCATs were synthesized using a wheat germ cell-free synthesis system. **c** SRM chromatogram of standard peptides (total 79 peptides) obtained by biosynthesis of stable isotope-labeled QconCATs. The synthesized QconCATs were purified with 6xHis-tag and digested with trypsin. The peptide mixtures derived from QconCATs were subjected to liquid chromatography (LC)-SRM. Fibrinopeptide released from the C terminus of QconCATs due to trypsin digestion was used for the absolute quantification of the synthesized QconCATs using SRM (Additional file 8: Figure S4C)

Combining established assays with stable isotope-labeled peptides of known concentration (AQUA peptides [45]) as an internal standard enables the measurement of the absolute amounts of target proteins (Fig. 5a). In this study, the absolute amount of major TGC protein components (K4ASM0, K4CMI0, K4D062, and K4BNH6) was analyzed using the AQUA peptide labeled with ^13^C/^15^N at the C-terminal lysine residue. Based on the heavy (internal standard) and light (endogenous) peak area ratio in the SRM chromatograph, the absolute amounts of the target proteins were calculated. The estimated amounts of target TGC proteins contained in 100 trichomes, assuming that all four TGCs in the tomato trichome head were recovered without loss, are shown in Additional file 7: Figure S3.

When there are many target proteins to be quantified, a quantitative strategy using a concatemer of standard peptides called QconCAT can be effective [46–48]. In particular, the QconCAT series that covers a group of target proteins categorized based on their biological functions will be useful for functional proteomic analyses. In this study, we synthesized QconCATs covering 50 TGC proteins, involved in the synthesis of secondary metabolites and observed in our proteomics analysis (Fig. 5b and Additional file 8: Figure S4). Only two types of QconCAT (SOL-01 and SOL-02) with a molecular weight of ~ 50 kDa were sufficient to cover all the peptides (Additional file 9: Table S5). The LC-SRM analysis using tryptic digests of the synthesized ^13^C/^15^N-labeled QconCATs revealed that 79 kinds of standard peptides covering all 50 target proteins were successfully synthesized (Fig. 5c).

## Discussion

MS-based proteomics using the shotgun technique has been used to successfully identify various TGC proteins in several plant species [49]. However, the complexity of the TGC proteome is high, and the detection of low-abundance components requires a large number of samples and complicated fractionated processing. In addition, the amount of data obtained by the analysis is enormous and is time-consuming to analyze. Therefore, a new analytical technique is required for quick and highly-sensitive quantitation of the protein component of interest in TGC samples. Herein, we proposed an experimental strategy for high-throughput acquisition of quantitative information on target TGC proteins using the high-sensitivity SRM assay.

The information of proteotypic peptides suitable for monitoring targeted proteins is essential for SRM assay development. In the human proteome, the peptides for several proteins have already been identified and their optimized SRM assay information is publicly available [50–52]. On the contrary, publicly available SRM information for plant proteomes is currently being developed rapidly [53], but its scale has so far been limited. In this study, we conducted a search for proteotypic peptides in the tomato TGC proteome using GeLC–MS/MS analysis. The combination of PAGE separation at the protein level and reversed-phase LC separation at the peptide level was effective for collecting a large amount of peptide information from complex TGC protein extracts. Using the TGC samples obtained from 800 trichomes, reliable SRM assay construction for detecting more than 300 major TGC proteins was possible in approximately 2 weeks. Although the established assay can cover high-abundance proteins in tomato TGCs, comprehensive quantification of the TGC proteome requires peptide information on low-abundance proteins. We used a triple quadrupole/linear ion trap hybrid MS system for GeLC–MS/MS analysis, but the use of a high-end MS instrument with faster scan-speed such as quadrupole time-of-flight MS [54] will improve the number of peptides detected. Sample pre-fractionation by introducing an additional separation technique such as isoelectric focusing electrophoresis will also be effective.

For type VI gland trichomes of tomato with four TGCs in the head, manual sampling using a pair of forceps was suitable for TGC sampling. Collection of TGCs after visual confirmation avoids sample contamination, which is a problem in the conventional large-scale recovery method of scraping and mesh-filtering [16]. In addition, our method can accurately count the number of harvested cells, which is useful to estimate the accurate protein expression level per cell. On the contrary, while sampling TGCs of small size, e.g., type I trichome of tomato, it may be effective to choose other approaches that are easier to operate than the use of forceps, such as absorbing cellular components on filter paper and aspirating with a capillary. For single cell metabolome analysis of tomato trichome, an example of recovering intracellular components using a pressure probe made of quartz capillary has been reported [55], and the application of such a method to TGC proteomics would also be interesting. The TGC sample (derived from 800 trichomes) recovered for GeLC–MS/MS is rich in essential oils, and it is preferable to purify protein components by methanol/chloroform precipitation [56]. Contrarily, a trace amount of TGC sample (derived from 50 to 100 glandular trichomes) prepared for the assay is readily solubilized by sonication in 50 μL of high concentration urea solution, and the oil in the sample had no influence on subsequent protease digestion treatment and peptide purification.

Protein quantification by the SRM assay has excellent detection sensitivity and wide dynamic range, which is suitable for simultaneously monitoring multiple targets from small biological samples in a single run. For example, the detection of the major TGC proteins by our established SRM assays was possible in 1/10th the amount of sample used for GeLC–MS/MS. The high detection sensitivity of SRM greatly contributes to the reduction in the amount of TGC used for analysis, allowing minimally invasive TGC sampling. For the measurement of 221 proteins, ~ 400 TGCs (derived from 100 glandular trichomes) recovered from a single tomato plant using a pair of forceps were sufficient, and the throughput performance of the analysis was greatly improved. Establishment of this SRM assay platform with high sensitivity and high throughput enables a reliable quantitative analysis of the TGC proteome using multiple samples.

Our SRM assays targeting 200 major proteins revealed that the proteomic profile of TGCs has significant differences at the organ level. For example, some proteins related to defense response to insects and pathogens (Q05540, K4B9P3, and Q7XBB3), cold and drought stress (K4BVU7), and reactive oxygen species (Q7YK44) were abundantly expressed in the leaves and/or calyx compared with those in the green fruits. Detailed spatiotemporal analysis of their expression levels in various environmental conditions is necessary to further understand their physiological role in each organ. To this end, multipoint monitoring of targeted proteins utilizing our high-throughput SRM-based approach will greatly contribute to speeding up and simplifying this analysis. Moreover, organ specificity was also observed in nine enzymes involved in secondary metabolite production (K4AS92, K4ASM0, K4C235, K4C6Q9, K4CJ96, K4CMI0, K4CW40, K4CYV4, and Q8GT30), which suggests the presence of inter-organ differences at the secondary metabolite level in TGCs. The development of a trans-omics workflow, which analyzes the metabolites contained in the TGC sample used for protein quantification simultaneously, is important for further understanding of the intracellular metabolic production process.

In MS-based protein quantification, stable isotope-labeled peptide standards are generally required to accurately know the absolute amount of the target proteins in a crude biological sample [57]. Our study demonstrated that the combination of SRM and AQUA peptides is a powerful approach for determining the absolute abundance of protein components in TGCs. However, one of the barriers in the use of this approach for targeted proteomics is the high cost involved in the preparation of AQUA peptides. To overcome this problem, the QconCAT strategy can be a powerful alternative for the cost-effective generation of reference peptides [58, 59]. In this study, we demonstrated that two stable isotope-labeled QconCAT proteins could produce 79 standard peptides, permitting the quantification of 50 proteins. Using a similar approach, the development of standard libraries for global quantification of all proteins identified in this study can be achieved by creating 6–8 QconCATs. Further expansion of QconCAT resources will greatly contribute to the realization of global proteome quantification in TGCs.

## Conclusions

In conclusion, a large-scale LC-SRM assay enabled high-throughput analysis of multiple target proteins contained in TGCs, which will be a useful tool for the high-throughput quantification of proteome dynamics in biological processes. Although the experimental workflow for assay development in this study targeted the TGC proteome, a similar approach would be applicable for targeted proteome analysis in other micro-tissues of a plant. Our assay information can be accessed via a public repository server PanoramaWeb [60] (https://panoramaweb.org/tomatotgc.url), which has been developed to support the sharing of SRM assay information and measurement data. Additional sequence information, including sequence homology of the targeted peptides in other plant species (Additional file 10: Figure S5), for assay development is available from our online reference database Ehime University SRM/MRM Reference Database (ESRDB) [58] (http://esrdb.m.ehime-u.ac.jp/index.html).

## Methods

### Reagents

Acetonitrile, acrylamide, ammonium bicarbonate (ABC), chloroform, methanol, trifluoroacetic acid (TFA), formic acid (FA), acetic acid, pure water, and sequencing grade Lys-C were obtained from Wako (Osaka, Japan). Urea was purchased from GE Healthcare (Pittsburg, PA, USA). Sequencing grade trypsin was purchased from Promega (Madison, WI, USA). Dithiothreitol (DTT) was purchased from Thermo Fisher Scientific (Waltham, MA, USA). Stable isotope-labeled AQUA peptides (99% purity) as an internal standard for protein quantification were obtained from Sigma-Aldrich (St. Louis, MO, USA). Artificially synthesized QconCAT genes were obtained from Eurofins Genomics (Tokyo, Japan).

### Plant material

The tomato cultivar, Micro-Tom (*S. lycopersicum* L. ‘Micro-Tom’), was used in the study. All the plants were grown on a growing medium consisting of peat moss (Sakata Seed Corp., Kanagawa, Japan) and incubated in a growth chamber (MLR-352H, Panasonic Corp., Osaka, Japan) maintained at air temperature, relative humidity, photosynthetic photon flux density, and photoperiod of 28 °C, 60%, 100 μmol/m^2^/s, and 16 h, respectively. Sufficient water and nutrient solutions were supplied periodically as described previously [55]. Trichome glandular cells of type VI glandular trichomes on the leaves, unripe green fruits, and calyx of approximately 8–12-week-old plants were collected with five biological replications and used in the subsequent analyses.

### Gel electrophoresis

Tomato TGCs were manually harvested using a pair of forceps from type VI glandular trichomes of tomato (n = 800) under a stereoscopic microscope and were collected in 50 μL SDS lysis buffer (4% [w/v] SDS in 150 mM Tris–HCl, pH 8.8). After homogenization using a BioMasher disposable homogenizer tube (Nippi, Tokyo, Japan), the extracted proteins were purified by methanol/chloroform precipitation. The precipitated proteins were solubilized in 10 μL of NuPAGE LDS sample buffer (Thermo Fisher Scientific) containing 200 mM DTT and subjected to electrophoretic separation using a NuPAGE 4–12% Bis–Tris gel (Thermo Fisher Scientific) in NuPAGE MOPS SDS running buffer (Thermo Fisher Scientific). The separated proteins were stained with BioSafe Coomassie brilliant blue (BioRad, Hercules, CA, USA) and the stained gel images were captured using a GELSCAN transmission scanner (iMeasure Inc., Nagano, Japan).

### In-gel protein digestion

A CBB-stained sample lane in the NuPAGE gel was divided into 16 pieces, and the cut gel pieces were used for in-gel trypsin digestion. Each gel piece was incubated with 100 μL of 50% (v/v) acetonitrile/50-mM ABC at 23 °C for 3 h to remove the CBB dye. The de-stained gel was incubated with 50 μL of 100 mM ABC/4 mM DTT at 37 °C for 1.5 h, and then incubated with 50 μL of 100 mM ABC/25 mM acrylamide at 23 °C for 30 min. The gel was washed with 500 μL of washing solution A (50% [v/v] methanol, 5% [v/v] acetic acid) for 1 h and further washed with 500 μL of washing solution B (50 mM ABC, 50% [v/v] acetonitrile). After washing, the gel was dehydrated with 200 μL of acetonitrile for 15 min, and then air-dried for 15 min. For in-gel protein digestion, 1 μL of trypsin solution (0.1 μg/μL) was added to the dry gel and incubated with 50 μL of 100 mM ABC at 37 °C for 16 h. The digested peptides in the gel were extracted by shaking it with 50 μL of 5% (v/v) TFA/50% (v/v) acetonitrile for 10 min. The extracted peptides were dried by vacuum centrifugation and resuspended in 10 μL of 0.1% TFA for the LC–MS/MS analysis.

### LC–MS/MS analysis

For the LC–MS/MS analysis of in-gel digested peptides, a system consisting of an Eksigent nanoLC system (SCIEX, Framingham, MA, USA) connected online to a Q-Trap 5500 hybrid triple quadrupole/linear ion trap mass spectrometer (SCIEX) was used. The peptide solution (5 μL) was injected into an Eksigent 200 µm i.d. × 0.5 mm cHiPLC trap column (SCIEX). The trapped peptides were then separated on a 75-µm i.d. × 15 cm C18 reversed phase cHiPLC column (SCIEX). For LC gradient separation, the mobile phase consisted of an aqueous solution of 0.1% FA as solvent A and 80% acetonitrile containing 0.1% FA and water as solvent B. The flow rate was 300 μL/min. The following gradient was used: 0–60 min, 2–18% B; 60–95 min, 18–40% B; hold at 90% B for 10 min, and equilibrate at 2% B for 15 min prior to the next run. The column temperature was maintained constant at 45 °C. The MS/MS data of the eluted peptides were acquired in the positive ion mode by information-dependent acquisition workflow as described previously [58]. The MS/MS data were processed using ProteinPilot V4.0 (SCIEX) search engine for protein identification. Peptide identification was performed against entries from the UniProt *Solanum lycopersicum* reference proteome (Organism ID: 4081; download date: Jun 1, 2013) using the following parameters: cys alkylation, acrylamide; digestion, trypsin; processing parameters, biological modification; search effort, through ID. Protein identities were accepted using a 1% false discovery rate.

### In-solution protein digestion

Manually harvested TGCs from tomato glandular trichomes (n = 100) were placed in 50 μL of urea lysis solution (8 M urea in 50 mM ABC) in a 0.5-mL low-adsorption tube (Eppendorf, Hamburg, Germany) and were subjected to sonication in an ultrasonic bath at 20 °C for 30 s. The sample tube was immediately transferred to a deep freezer and stored at − 80 °C until in-solution digestion. After being mixed with 2 μL of 200 mM DTT, the sample solution was incubated at 37 °C for 1.5 h, followed by alkylation with 2 μL of 1.2 M acrylamide for 30 min at 23 °C. The proteins in the solution were digested with 0.2 μg of Lys-C for 4 h at 23 °C. After diluting the solution with 200 μL of 100 mM ABC, further digestion was carried out with 0.2 μg of trypsin for 12 h at 37 °C. The digested peptides were purified using a self-made SDB-XC STAGE tip. The obtained peptides were dried using vacuum centrifugation, resuspended in 10 μL of 0.1% (v/v) TFA, and subjected to LC-SRM analysis. For each run, 5 μL of sample was injected.

### LC-SRM assay

The development of the SRM assays with Skyline software was performed as described previously [58]. The database search files from ProteinPilot software (SCIEX) were used to construct the MS spectral library containing only tryptic digested peptides (allowed one missed cleavage) with a confidence value of > 0.9. Up to five peptides of length 6–30 amino acids were selected per protein for the SRM analysis. SRM transitions were selected from the 3 to 4 most intense y and b product ions among + 2 or + 3 charged precursors of each peptide. The LC-SRM assay was performed using the same LC–MS system (SCIEX QT5500) used for the GeLC–MS/MS analysis. Analysis of the obtained SRM data was performed with Skyline. Endogenous peptide peaks in the acquired SRM chromatogram were selected manually based on the chromatographic elution pattern and dot product (dotp) value.

### Protein quantification using the AQUA peptide

Tomato TGCs derived from 100 type VI trichomes were digested in the urea lysis solution, as described above, and the obtained digests were mixed with 5 μL of AQUA peptide solution (10 fmol/μL). After purification of the peptide sample with a STAGE tip, the target peptides in the sample were detected by SRM. The absolute amount of the target endogenous protein was estimated by comparing the light and heavy peak areas of the selected monitoring peptide.

### Cell-free synthesis of heavy-labeled QconCATs

Wheat germ cell-free synthesis of QconCAT proteins was performed using the WEPRO8240H expression kit (Cell-Free Sciences, Matsuyama, Japan) as described previously [59]. In vitro translation of the QconCAT proteins labeled with L-Arg-^13^C_6_,^15^N_4_, L-Lys-^13^C_6_, and ^15^N_2_ was performed in a bilayer system (200 μL of substrate layer and 40 μL of translation layer) at 17 °C for 20 h. The synthesized QconCAT proteins were purified using Ni-Sepharose High-Performance resin (GE Healthcare Life Sciences).

### Statistical analysis

The PCA and *t*-tests were performed using MarkerView software ver. 1.2.1 (SCIEX).

## Additional files

**Additional file 1: Figure S1.** Protein assay of trichome glandular cell (TGC) samples derived from a type VI trichome of tomato. (A) Experimental workflow. The total amount of protein extracted from the TGC sample was analyzed using the Qubit protein assay kit (Thermo Fisher Scientific), according to the manufacturer’s instructions. (B) The estimated amount of the total TGC protein derived from a single trichome from different organs (the fruits, leaves, and calyx). Bars represent the mean ± SD of four biological replicates.

**Additional file 2: Table S1.** Tomato trichome glandular cell (TGC) proteins identified by gel electrophoresis-assisted liquid chromatography-tandem mass spectrometry (GeLC–MS/MS) analysis. TGC proteins were identified using ProteinPilot (SCIEX) software with a false discovery rate (FDR) of 1%. Accession: accession ID in UniProtKB database; Protein Name: name of identified protein; Identified Peptide #: number of tryptic peptide identified by MS/MS, the cut-off for peptide confidence scores was > 95;  % Sequence Coverage: the percentage of the protein sequence covered by identified peptides with confidence score > 95.

**Additional file 3: Figure S2.** Shotgun proteomics of tomato trichome glandular cells (TGCs) using gel electrophoresis-assisted liquid chromatography-tandem mass spectrometry (GeLC–MS/MS). (A) Biological processes of the TGC proteins identified by GeLC–MS/MS. Gene ontology analysis using PANTHER 14.0 (http://pantherdb.org) was performed on the proteins identified at FDR 1%. (B) Metabolic pathways related to the proteins identified by shotgun proteomics are highlighted in the Kyoto Encyclopedia of Genes and Genomes (KEGG) pathway map (https://www.genome.jp/kegg/pathway.html).

**Additional file 4: Table S2.** Established selected reaction monitoring (SRM) assays targeting tomato trichome glandular cell (TGC) proteome. The SRM assay was developed using Skyline Software. Q1: quadrupole mass filter to select a precursor ion; Q3: quadrupole mass filter to select a fragment ion; Protein: protein targeted by the assay; Sequence: monitoring peptide sequence; Charge: charge of precursor ion selected by Q1; Fragment ion: fragment ion selected by Q3; DP: declustering potential; CE: collision energy; Verified: SRM transition which was verified using tryptic digest of tomato TGC extract.

**Additional file 5: Table S3.** Relative expression levels of targeted proteins in tomato trichome glandular cells (TGCs). The relative expression levels of target protein in each sample type (fruit, leaf, and calyx) was estimated based on the selected reaction monitoring (SRM) peak area of the corresponding peptide. Protein: targetedprotein name; Peptide: peptide sequence used for selected reaction monitoring (SRM) quantitative analysis.

**Additional file 6: Table S4.** Differential expression of trichome glandular cell (TGC) proteins between tomato organs. Fold Change: fold change for differential protein expression; *p* value: statistical significance; Accession: accession IDs of proteins with differential expression between different organs; Protein: targeted protein name; Peptide: peptide sequence used for selected reaction monitoring (SRM) quantitative analysis; GO - Molecular function: gene ontology annotation for molecular function; GO - Biological process: gene ontology annotation for biological process.

**Additional file 7: Figure S3.** Absolute quantification of targeted proteins in tomato trichome glandular cells (TGCs) using AQUA peptide. Tryptic peptides shown on the graphs were used for selected reaction monitoring (SRM) quantification.

**Additional file 8: Figure S4.** High-throughput generation of standard peptides for selected reaction monitoring (SRM) quantification using QconCAT strategy. (A) Experimental workflow of QconCAT biosynthesis using a wheat germ cell-free system. (B) Representative polyacrylamide gel electrophoresis (PAGE) images of synthesized QconCATs. For gel electrophoresis, 4%–12% NuPAGE gel was used. Separated QconCATs were visualized with CBB-R250. (C) Absolute quantification of the synthesized QconCATs. (D) SRM chromatograms of QconCATs. Tryptic digests from stable-isotope-labeled QconCATs (SOL-01 and SOL-02) were individually analyzed by liquid chromatography (LC)-SRM.

**Additional file 9: Table S5.** QconCAT information. Sequence: amino acid sequence of designed QconCAT; Molecular Weight: theoretical molecular weight of QconCAT; Synthesized Gene Sequence: nucleic acid sequence of artificial synthetic gene encoding QconCAT; Accession: accession ID of protein to be quantified by QconCAT; Peptide: peptide sequence used for QconCAT design; KEGG Information: information on enzymatic function from the Kyoto Encyclopedia of Genes and Genomes (KEGG) database (sly01110 Biosynthesis of secondary metabolites - *Solanum lycopersicum*).

**Additional file 10: Figure S5.** Ehime University selected reaction monitoring (SRM)/multiple reaction monitoring (MRM) reference database (ESRDB): online reference database for assays. Detailed sequence information including sequence homology of target peptides in *Solanum lycopersicum* and other plant species (*Arabidopsis thaliana, Nicotiana tabacum*, and *Artemisia annua*) is available.

## Abbreviations

TGC: trichome glandular cell
MS: mass spectrometry
SRM: selected reaction monitoring
LC: liquid chromatography
MS/MS: tandem MS
GeLC–MS/MS: gel electrophoresis-assisted LC–MS/MS
PAGE: polyacrylamide gel electrophoresis
SDS: sodium dodecyl sulfate
ABC: ammonium bicarbonate
DTT: dithiothreitol
FA: formic acid
TFA: trifluoroacetic acid
