# Supplementary material for: Quantitative assay of targeted proteome in tomato trichome glandular cells using a large-scale selected reaction monitoring strategy
Source: Plant Methods. 2019 Apr 24;15:40. doi: 10.1186/s13007-019-0427-7 (PMC6480907; doi:10.1186/s13007-019-0427-7)
Supplement: Supplementary file 1 — Additional file 1: Figure S1. Protein assay of trichome glandular cell (TGC) samples derived from a type VI trichome of tomato. (A) Experimental workflow. The total amount of protein extracted from the TGC sample was analyzed using the Qubit protein assay kit (Thermo Fisher Scientific), according to the manufacturer’s instructions. (B) The estimated amount of the total TGC protein derived from a single trichome from different organs (the fruits, leaves, and calyx). Bars represent the mean ± SD of four biological replicates. [file 13007_2019_427_MOESM1_ESM.pdf]

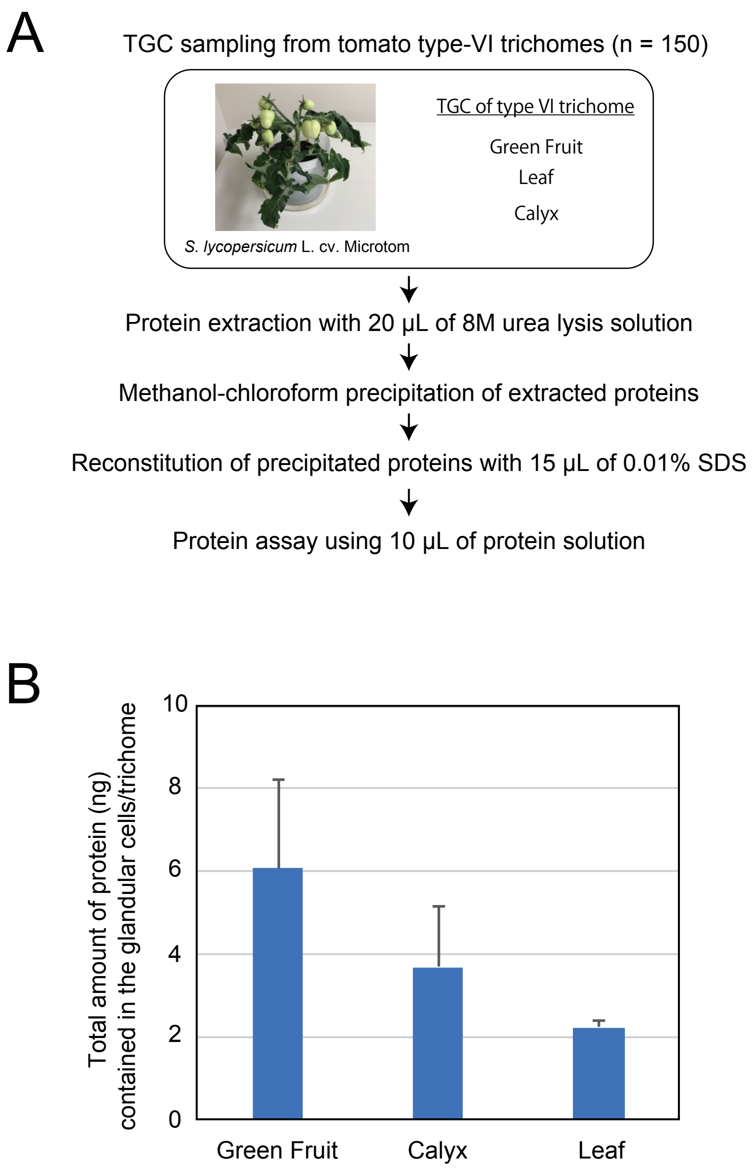
A
TGC sampling from tomato type-VI trichomes (n = 150)
TGC of type VI trichome
Green Fruit
Leaf
Calyx
S. lycopersicum L. cv. Microtom
Protein extraction with 20 μL of 8M urea lysis solution
Methanol-chloroform precipitation of extracted proteins
Reconstitution of precipitated proteins with 15 μL of 0.01% SDS
Protein assay using 10 μL of protein solution
B
Total amount of protein (ng) contained in the glandular cells/trichome
0
2
4
6
8
10
Green Fruit
Calyx
Leaf
