# Supplementary figures and images for: Quantitative assay of targeted proteome in tomato trichome glandular cells using a large-scale selected reaction monitoring strategy
Source: Plant Methods. 2019 Apr 24;15:40. doi: 10.1186/s13007-019-0427-7 (PMC6480907; doi:10.1186/s13007-019-0427-7)

A

## PANTHER GO-Slim Biological Process

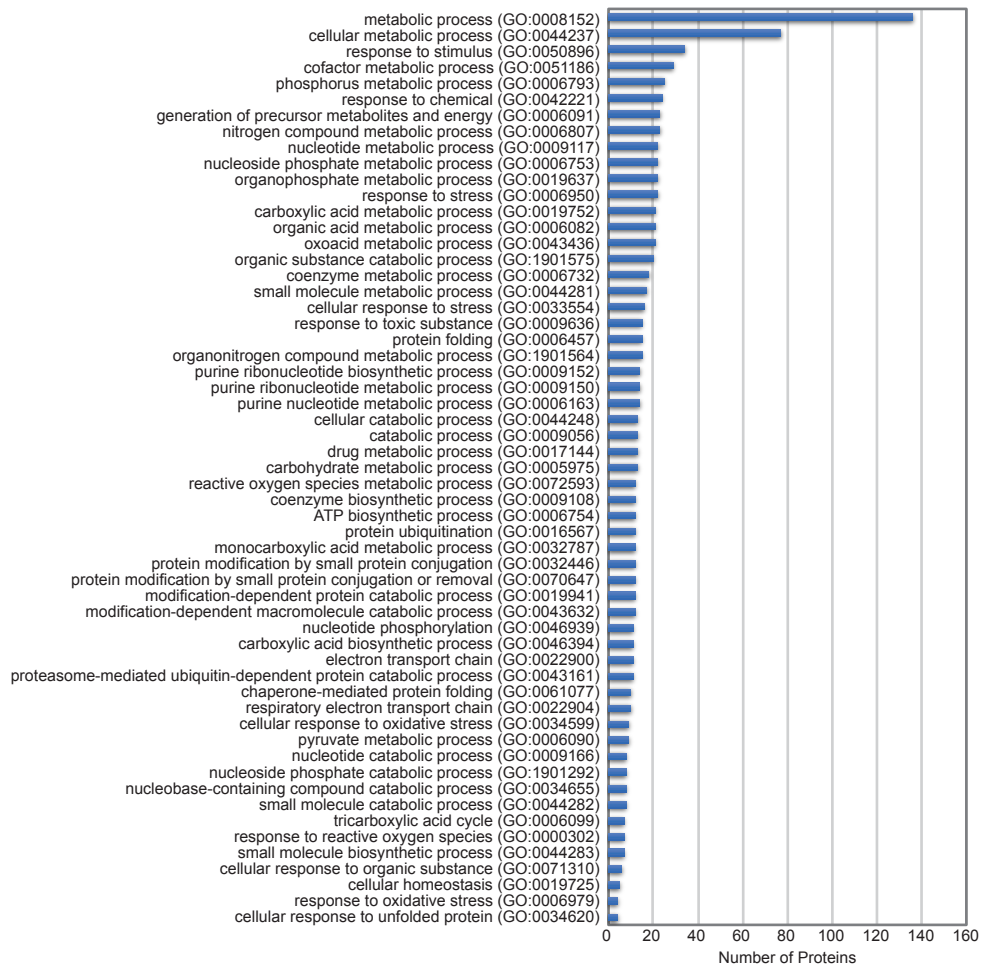

B

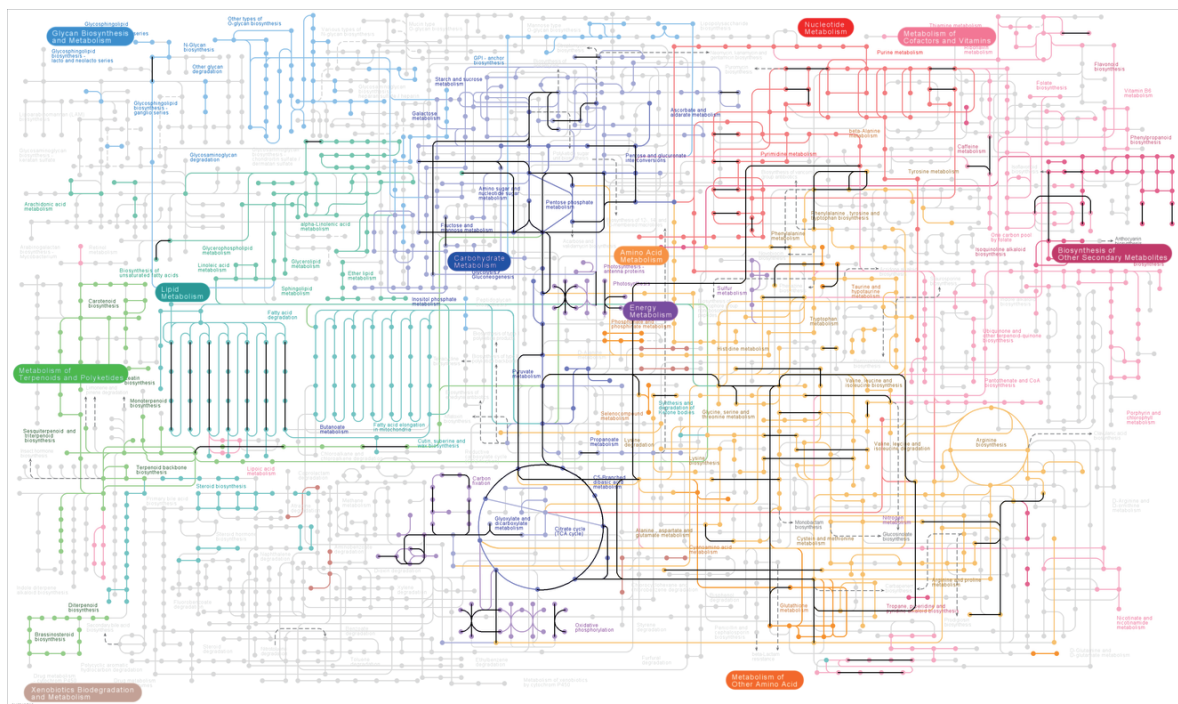

Supplement: Supplementary file 3 — Additional file 3: Figure S2. Shotgun proteomics of tomato trichome glandular cells (TGCs) using gel electrophoresis-assisted liquid chromatography-tandem mass spectrometry (GeLC–MS/MS). (A) Biological processes of the TGC proteins identified by GeLC–MS/MS. Gene ontology analysis using PANTHER 14.0 (http://pantherdb.org) was performed on the proteins identified at FDR 1%. (B) Metabolic pathways related to the proteins identified by shotgun proteomics are highlighted in the Kyoto Encyclopedia of Genes and Genomes (KEGG) pathway map (https://www.genome.jp/kegg/pathway.html). [file 13007_2019_427_MOESM3_ESM.pdf]

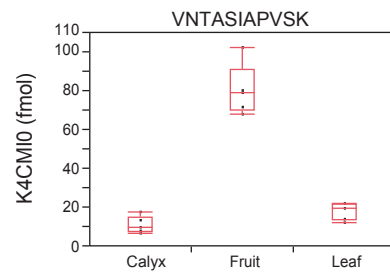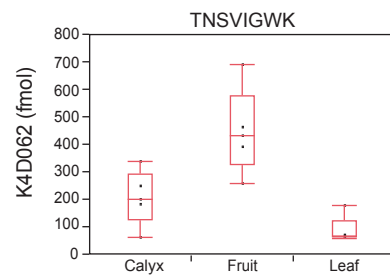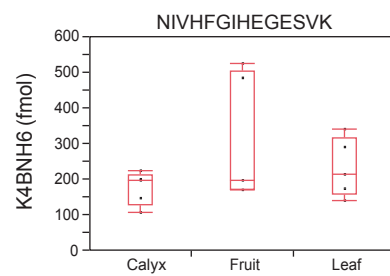

Supplement: Supplementary file 7 — Additional file 7: Figure S3. Absolute quantification of targeted proteins in tomato trichome glandular cells (TGCs) using AQUA peptide. Tryptic peptides shown on the graphs were used for selected reaction monitoring (SRM) quantification. [file 13007_2019_427_MOESM7_ESM.pdf]

**A**

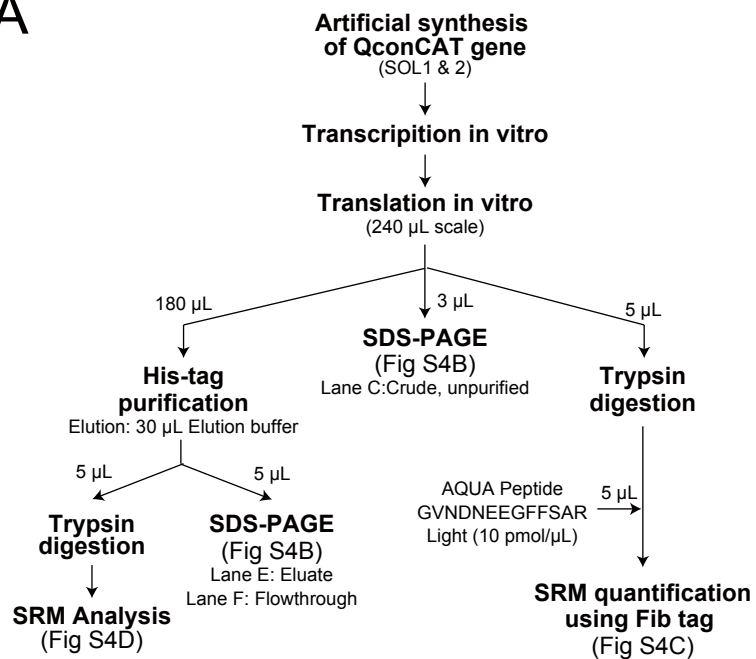

**B**

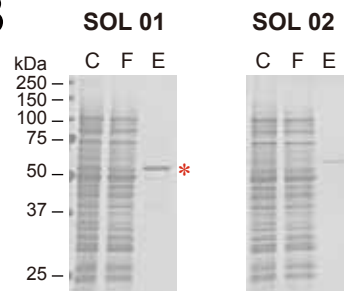

**C**

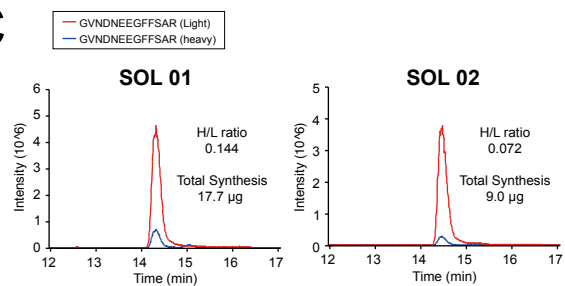

**D**

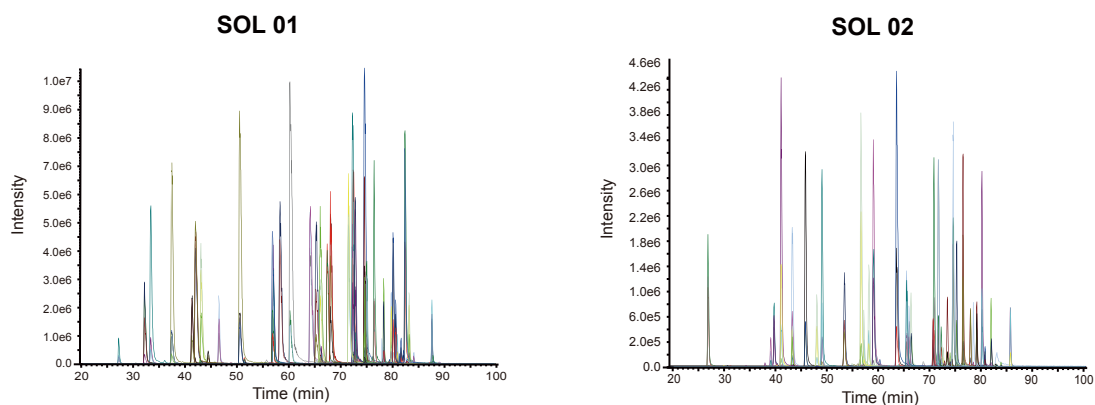

Supplement: Supplementary file 8 — Additional file 8: Figure S4. High-throughput generation of standard peptides for selected reaction monitoring (SRM) quantification using QconCAT strategy. (A) Experimental workflow of QconCAT biosynthesis using a wheat germ cell-free system. (B) Representative polyacrylamide gel electrophoresis (PAGE) images of synthesized QconCATs. For gel electrophoresis, 4%–12% NuPAGE gel was used. Separated QconCATs were visualized with CBB-R250. (C) Absolute quantification of the synthesized QconCATs. (D) SRM chromatograms of QconCATs. Tryptic digests from stable-isotope-labeled QconCATs (SOL-01 and SOL-02) were individually analyzed by liquid chromatography (LC)-SRM. [file 13007_2019_427_MOESM8_ESM.pdf]
