# Supplementary material for: Quantitative assay of targeted proteome in tomato trichome glandular cells using a large-scale selected reaction monitoring strategy
Source: Plant Methods. 2019 Apr 24;15:40. doi: 10.1186/s13007-019-0427-7 (PMC6480907; doi:10.1186/s13007-019-0427-7)
Supplement: Supplementary file 10 — Additional file 10: Figure S5. Ehime University selected reaction monitoring (SRM)/multiple reaction monitoring (MRM) reference database (ESRDB): online reference database for assays. Detailed sequence information including sequence homology of target peptides in Solanum lycopersicum and other plant species (Arabidopsis thaliana, Nicotiana tabacum, and Artemisia annua) is available. [file 13007_2019_427_MOESM10_ESM.pdf]

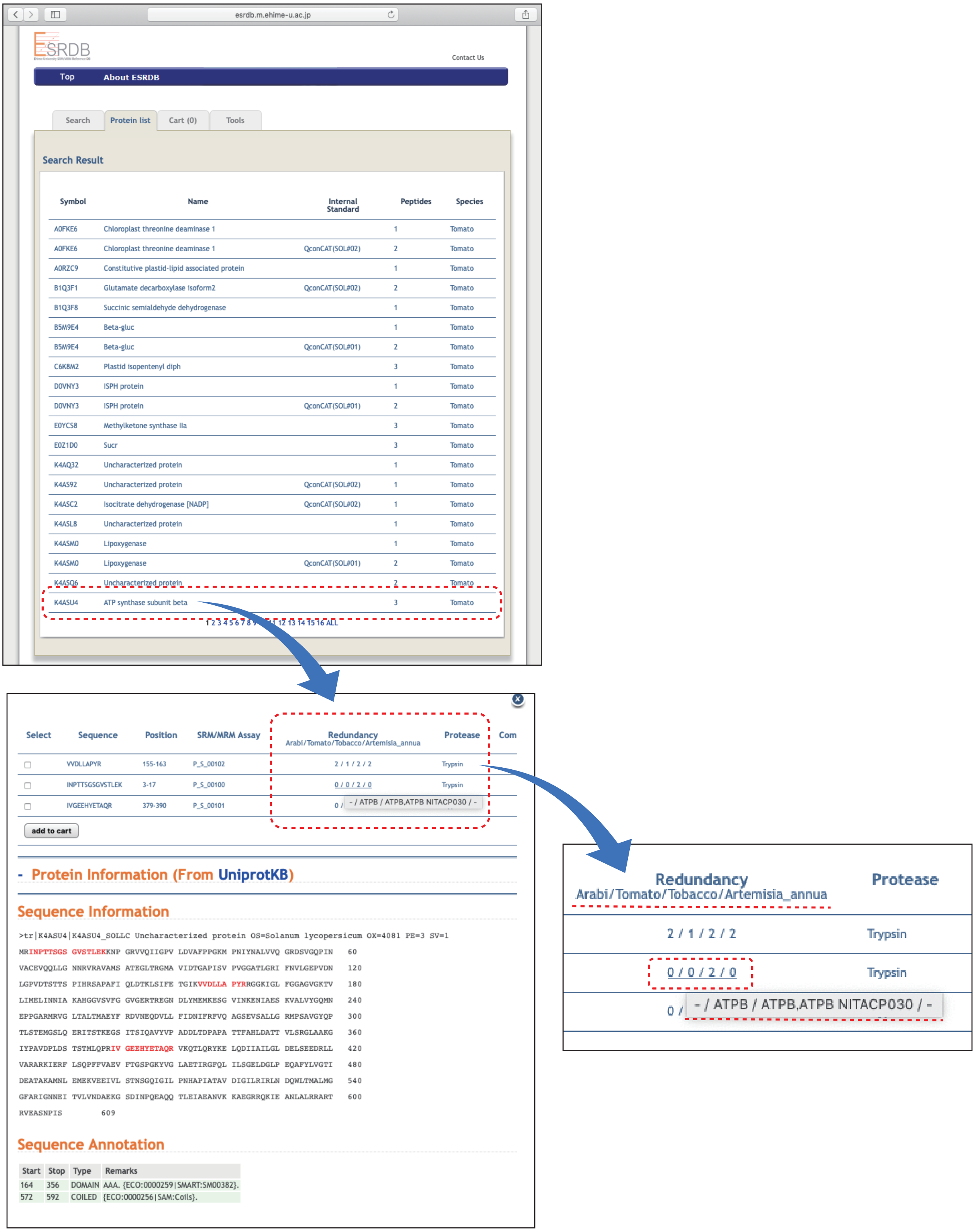

esrdb.m.ehime-u.ac.jp
ESRDB
Contact Us
Top
About ESRDB
Search
Protein list
Cart (0)
Tools
Search Result
Symbol | Name | Internal Standard | Peptides | Species
A0FKE6 | Chloroplast threonine deaminase 1 | | 1 | Tomato
A0FKE6 | Chloroplast threonine deaminase 1 | QconCAT(SOL#02) | 2 | Tomato
A0RZC9 | Constitutive plastid-lipid associated protein | | 1 | Tomato
B1Q3F1 | Glutamate decarboxylase isoform2 | QconCAT(SOL#02) | 2 | Tomato
B1Q3F8 | Succinic semialdehyde dehydrogenase | | 1 | Tomato
B5M9E4 | Beta-gluc | | 1 | Tomato
B5M9E4 | Beta-gluc | QconCAT(SOL#01) | 2 | Tomato
C6K8M2 | Plastid isopentenyl diph | | 3 | Tomato
D0VNY3 | ISPH protein | | 1 | Tomato
D0VNY3 | ISPH protein | QconCAT(SOL#01) | 2 | Tomato
E0YCS8 | Methylketone synthase IIa | | 3 | Tomato
E0Z1D0 | Sucr | | 3 | Tomato
K4AQ32 | Uncharacterized protein | | 1 | Tomato
K4AS92 | Uncharacterized protein | QconCAT(SOL#02) | 1 | Tomato
K4ASC2 | Isocitrate dehydrogenase [NADP] | QconCAT(SOL#02) | 1 | Tomato
K4ASL8 | Uncharacterized protein | | 1 | Tomato
K4ASM0 | Lipoxygenase | | 1 | Tomato
K4ASM0 | Lipoxygenase | QconCAT(SOL#01) | 2 | Tomato
K4ASQ6 | Uncharacterized protein | | 2 | Tomato
K4ASU4 | ATP synthase subunit beta | | 3 | Tomato
Select | Sequence | Position | SRM/MRM Assay | Redundancy Arabi/Tomato/Tobacco/Artemisia_annua | Protease | Com
VVDLLAPYR | 155-163 | P_S_00102 | 2 / 1 / 2 / 2 | Trypsin
INPTTSGSGVSTLEK | 3-17 | P_S_00100 | 0 / 0 / 2 / 0 | Trypsin
IVGEEHYETAQR | 379-390 | P_S_00101 | 0 /
- / ATPB / ATPB,ATPB NITACP030 / -
add to cart
- Protein Information (From UniprotKB)
Sequence Information
>tr|K4ASU4|K4ASU4_SOLLC Uncharacterized protein OS=Solanum lycopersicum OX=4081 PE=3 SV=1
MRINPTTSGS GVSTLEKKNP GRVVQIIGPV LDVAFPPGKM PNIYNALVVQ GRDSVGQPIN 60
VACEVQQLLG NNRVRAVAMS ATEGLTRGMA VIDTGAPISV PVGGATLGRI FNVLGEPVDN 120
LGPVDTSTTS PIHRSAPAFI QLDTKLSIFE TGIKVVDLLA PYRRGGKIGL FGGAGVGKTV 180
LIMELINNIA KAHGGVSVFG GVGERTREGN DLYMEMKESG VINKENIAES KVALVYGQMN 240
EPPGARMRVG LTALTMAEYF RDVNEQDVLL FIDNIFRFVQ AGSEVSALLG RMPSAVGYQP 300
TLSTEMGSLQ ERITSTKEGS ITSIQAVYVP ADDLTDPAPA TTFAHLDATT VLSRGLAAKG 360
IYPAVDPLDS TSTMLQPRIV GEEHYETAQR VKQTLQRYKE LQDIIAILGL DELSEEDRLL 420
VARARKIERF LSQPFFVAEV FTGSPGKYVG LAETIRGFQL ILSGELDGLP EQAFYLVGTI 480
DEATAKAMNL EMEKVEEIVL STNSGQIGIL PNHAPIATAV DIGILRIRLN DQWLTMALMG 540
GFARIGNNEI TVLVNDAEKG SDINPQEAQQ TLEIAEANVK KAEGRRQKIE ANLALRRART 600
RVEASNPIS 609
Sequence Annotation
Start | Stop | Type | Remarks
164 | 356 | DOMAIN | AAA. {ECO:0000259|SMART:SM00382}.
572 | 592 | COILED | {ECO:0000256|SAM:Coils}.
Redundancy
Arabi/Tomato/Tobacco/Artemisia_annua
Protease
2 / 1 / 2 / 2
Trypsin
0 / 0 / 2 / 0
Trypsin
0 /
- / ATPB / ATPB,ATPB NITACP030 / -
